# Comparison of Antibacterial Activity and Wound Healing in a Superficial Abrasion Mouse Model of *Staphylococcus aureus* Skin Infection Using Photodynamic Therapy Based on Methylene Blue or Mupirocin or Both

**DOI:** 10.3389/fmed.2021.673408

**Published:** 2021-05-25

**Authors:** Montserrat Pérez, Pilar Robres, Bernardino Moreno, Rosa Bolea, Maria T. Verde, Vanesa Pérez-Laguna, Carmen Aspiroz, Yolanda Gilaberte, Antonio Rezusta

**Affiliations:** ^1^Animal Pathology Department, Veterinary Faculty, Zaragoza University, Zaragoza, Spain; ^2^Department of Microbiology, Hospital de Barbastro, Huesca, Spain; ^3^Department of Microbiology, Hospital Universitario Miguel Servet, IIS Aragón, Zaragoza, Spain; ^4^Department of Microbiology, Hospital Royo Villanova, IIS Aragón, Zaragoza, Spain; ^5^Department of Dermatology, Hospital Universitario Miguel Servet, IIS Aragón, Zaragoza, Spain

**Keywords:** *S aureus*, SKH-1 mice, superficial wound infection, wound healing, photoinactivation, mupirocin, antimicrobial

## Abstract

**Background:** Antibiotic resistance and impaired wound healing are major concerns in *S. aureus* superficial skin infections, and new therapies are needed. Antimicrobial photodynamic therapy (aPDT) is a new therapeutic approach for infections, but it also improves healing in many wound models.

**Objective:** To compare the antimicrobial activity and the effects on wound healing of aPDT based on Methylene Blue (MB-aPDT) with mupirocin treatment, either alone or in combination, in superficial skin wounds of *S. aureus*-infected mice. Additionally, to evaluate the clinical, microbiological, and cosmetic effects on wound healing.

**Materials and Methods:** A superficial skin infection model of *S. aureus* was established in SKH-1 mice. Infected wounds were treated with MB-aPDT, MB-aPDT with a daily topical mupirocin or only with mupirocin. No treatment was carried out in control animals. Daily clinical and microbiological examinations were performed until complete clinical wound healing. Histopathological studies and statistical analysis were performed at the end of the study.

**Results:** MB-aPDT treatment induced the best wound healing compared to mupirocin alone or to mupirocin plus MB-aPDT. Superficial contraction at 24 h and a greater reduction in size at 48 h, quicker detachment of the crust, less scaling, and absence of scars were observed. Histopathological studies correlated with clinical and gross findings. By contrast, mupirocin showed the highest logaritmic reduction of *S. aureus*.

**Conclusions:** MB-aPDT and mupirocin treatments are effective in a murine superficial skin infection model of *S. aureus*. One session of MB-aPDT was the best option for clinical wound healing and cosmetic results. The addition of mupirocin to MB-aPDT treatment improved antimicrobial activity; however, it did not enhance wound healing. No synergistic antibacterial effects were detected.

## Resume

Antimicrobial photodynamic therapy based on Methylene Blue (MB-aPDT) is recognized for wound healing and microbial properties. To compare *in vivo* efficacy of MB-aPDT vs. mupirocin (MU), we utilized a murine superficial abrasive wound model to study bacterial count, wound healing and cosmetics results. Mice were wounded dorsally and infected with *Staphylococcus aureus* (ATCC 29213 strain). Experimentally infected wound was treated topically either with MB-aPDT, MU, MB-aPDT+MU, or left untreated (control). Topically wounds were daily monitored (bacterial burden and wound healing) until clinical resolution. All mice develop purulent wounds as result of infection. Clinical evolution, gross observations, and histopathological findings are representative of acute infection, dermal response and histopathological hyperkeratosis at the clinical cure in animal model and antimicrobial trial. We demonstrated benefits of treatment in relation to control wounds. This study suggests that both treatments are significantly effective: MB-aPDT improves quick mild wound contraction at 24 h, better wound healing (reduction of size, crust loss) and cosmetics results (no scar). MU enhances antimicrobial activity which seems not to be relevant for wound healing. Best clinical healing was observed in wounds treated with MB-aPDT but further studies were warranted to test the effectiveness of more sessions of MB-aPDT to enhance microbicide formulation of aPDT, alone or in combination with antibiotics. No antimicrobial synergistic effect was observed in this self-limiting infection model. Further experiments may be a suitable choice.

## Introduction

Staphylococcus spp. causes 90% of torpid wound healing skin infection (1). *Staphylococcus aureus* is present in 60% of the biofilms of chronic wounds (2, 3), in the skin of 90% atopic patients (4) and it causes 75% of primary human pyoderma (5). Moreover, it is highlighted that one fifth of skin and soft tissue infections patients that received antibiotics develop recurrent skin infection with the same strain of *S. aureus*, developing resistance and increase of the dose of antimicrobials needed (6–8). Regarding mice, *S. aureus* is ubiquitous in the digestive and nasal mucosa and causes purulent dermatoses (9); additionally, in skin infection model, it mimics human disease (10) with suppurative dermatitis and abscesses (11) in mice SKH-1 (12, 13).

In this context, murine infection *S. aureus* models are recommended for *in vivo* development of new antimicrobials. Different types of incisions (scalpel, punch), *tape stripping* o burning procedures promotes skin sensibility to diverse types of bacteria, although *S. aureus* is able to cause infection even in intact skin (14). There is not an ideal model and it is desirable to select the best choice for each purpose (15). Cutaneous procedures are easily in hairless mice and histopathology has typical features that reproduces cutaneous responses of humans (16, 17). On the other hand, skin contraction mainly in full thickness wound model is problematic (18) and wound healing in superficial model was developed by re-epithelization instead of tissue granulation (19). A superficial *S. aureus* infection model, better using abrasion that *tape stripping*, have difficulties due to self-limiting course and quick resolution in maximum 8 days (20). This model requires high bacterial load to infect the wound bed and social isolation of mice previous acclimation, however, it has been validated to evaluate topical antimicrobial therapies (21, 22). SKH-1 hairless mice have been validated for dermatological studies and it is recommended for wound healing studies (23).

According to guidelines, mupirocin (MU) is one of the main topical treatments for *S. aureus* skin infections and also in risk of developing resistances (24, 25). On the other hand, facing the problem of antimicrobial resistance, one of the biggest challenges in medicine is to find alternative therapeutic treatments (8). Antimicrobial photodynamic therapy (aPDT) is a promising treatment for skin and mucosal infections whose mechanism of action is effective regardless of the antimicrobial resistance pattern (26). It is based on photosensitizer molecules with the propriety of being activated by visible light and react with oxygen generating reactive oxygen species, toxic to target cells (27). aPDT based on Methylene Blue (MB, MB-aPDT), the principal photosensitizer of the phenothiazine family, is a low-cost and easy-to-use technique that has already shown its antimicrobial applications in periodontal disease, impetigo or the exacerbation of human atopic dermatitis and the treatment cutaneous mycoses, leishmaniasis, and infected wounds (27–31) used in animals (32) and skin *S. aureus* murine models (33–35).

Among the drawbacks of aPDT, the main concern is the possibility of microbial regrowth after aPDT. Multiple sessions of aPDT or the combination of aPDT with topical antibiotic may avoid bacterial regrowth after it (even at 24 h) (20, 36–38).

Our group demonstrated the synergistic bactericidal effect of the combination of MB-aPDT with the antibiotics gentamicin, linezolid, or MU against *in vitro S. aureus* being the combination with the latter the most promising to transfer to the clinic (39, 74). However, *in vitro* results frequently overestimate *in vivo* findings (40). Factors of animal model such as individual microbiome or virulence are determinants (41, 42).

Here, this *in vivo* study compares the antimicrobial efficacy and skin regenerative effects (wound healing and cosmetics results) of MB-aPDT, topical MU or their combination in wounds infected with *S. aureus* of SKH-1 mice. Additionally, we optimize a superficial model of skin *S. aureus* infection in terms of macroscopic (gross aspects) and cosmetic result, histological findings, besides microbial counts for a therapeutical challenge.

## Materials and Methods

### Animals

SKH-1 hairless mice were obtained from the Charles River Laboratories (Germany). All mice were 6–8-week-old females and were individually kept in cages for a few days prior to the experiment to acclimate and throughout the experiment to prevent wound damage. Cages were placed close to the ground for mice to acclimate to low light levels and mice were provided with commercial feed and water ad libitum. All procedures were carried out in biosafety chambers (LAF, Laminar Air Flow) for type 2 pathogens, located at the Centro de Investigación Biomédica de Aragón (CIBA, Zaragoza, Spain). Mice were anesthetized by inhalation with 2% isofluorane.

All experimental procedures performed with animals were approved by the Ethic Committee for Animal Experiments from the University of Zaragoza (PI40/13).

### Bacterial Strains

Methicillin-sensible *S. aureus* ATCC 29213 strain was obtained from the American Type Culture Collection (ATCC, Rockville, MD, USA). Bacteria were grown aerobically overnight on blood agar plates at 35°C and the inoculum was prepared following the standard procedures at the Microbiology Department of the Zaragoza Medical School. The inoculum was prepared adding colonies in distilled water (Gibco®, Thermofisher, Spain) and adjusted to 0.50 ± 0.03 on the McFarland scale. A final concentration of 3 × 10^8^ CFU/mL was obtained.

### Superficial Skin Infection and Microbiological Evaluation

The *in vivo* model infection was colonization incisional by abrasion (20, 34) with a dose infective (3 × 108 colony forming units (CFU)/mL) growing from 4 first hours in a self-limiting pattern (14) with bacterial maturation (105 CFU/mL) in 48 h post-inoculation and clinical duration of 10–12 days.

**Figure 1:**
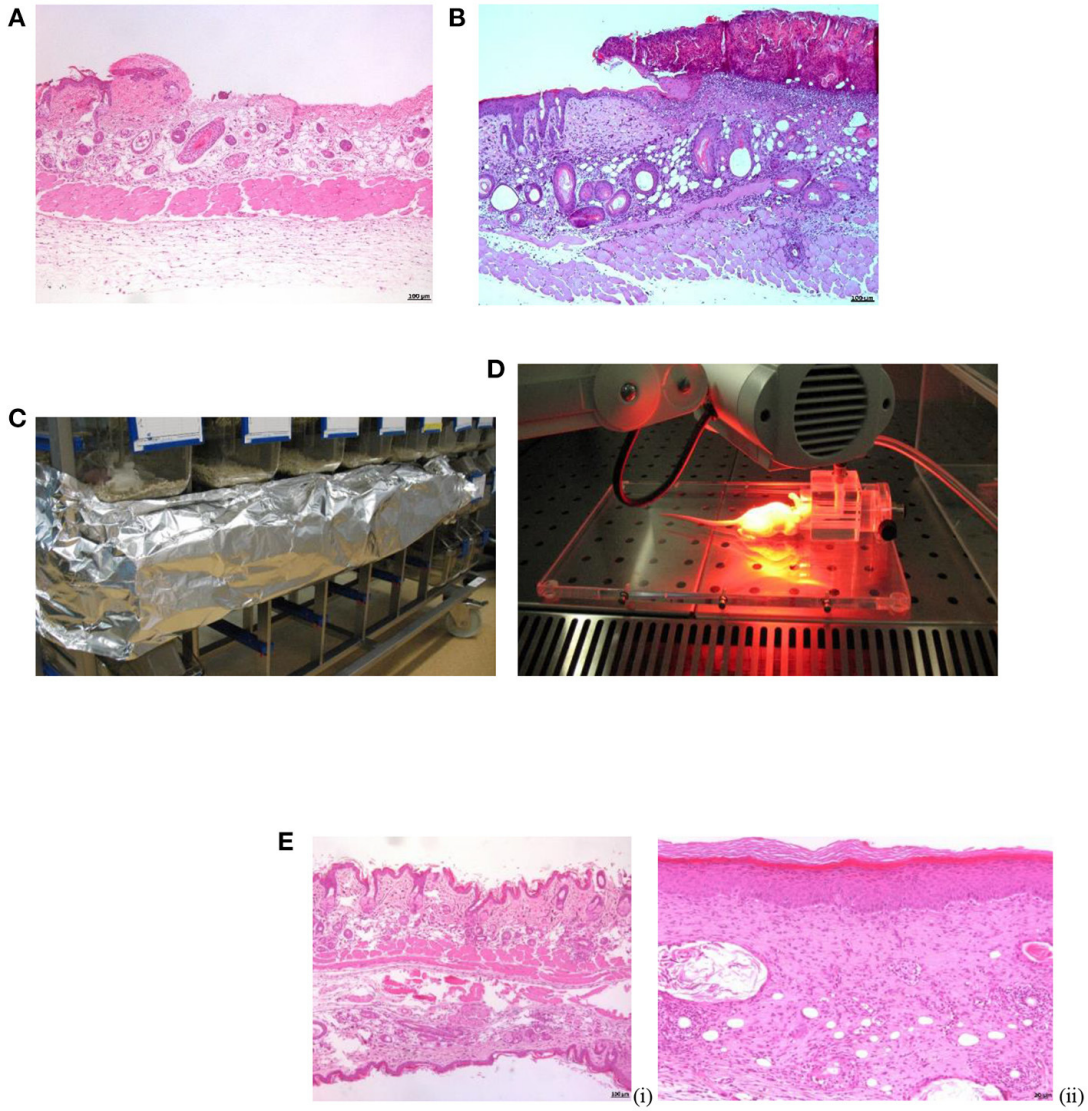
Procedures in SKH-1 mice. **(A)** Microphotograph of skin abrasion experimentally induced. Note the very superficial loss of the epidermis. H-E. x50. **(B)** Microphotograph of the purulent crust observed at 24–48 h post-infection. H-E. x50. **(C)** Incubation of BM (aPDT) on dark. **(D)** MBa-PDT session. **(E)** Microphotograph of SKH-1 mice healthy skin (i) vs. skin healed per se (S. aureus infection) at 13 days post-inoculation (ii). H-E. x50.

Two superficial abrasions were made in 20 mice under general anesthesia (dexmedetomidin/Ketamine IP (1 mg/kg + 75 mg/kg, Dexdomitor® 0.1 mg/mL + Imalgene® 50 mg/mL) mice (*n* = 20). Skin was disinfected with 70% alcohol and abrasions were carried out with a scalpel (n° 11) until redness appeared and epidermis apparently was lost (Figure 1A). Wounds were ~0.6 cm in diameter and were located in the dorsal area and at a distance of 1 cm. Wounds were infected with the inoculum previously obtained and protected with a transparent sticking plaster (Omnifilm®, Hartmann) for 24 h. Mice were euthanized with CO_2_ when wounds were healed and skin biopsies were taken. To minimize the number of animals used in the experiment, each mouse has a wound used as its own control (43).

To determine the bacterial burden of the wounds throughout the experiment, swabs (DeltaSwab Amies®, DeltaLab, Spain) were taken daily. To avoid contamination, swabs were taken prior to any procedure. Samples were studied using routine microbiological procedures. They were plated on sheep blood agar (no selective) and CNS agar (selective for coagulase negative staphylococci) and the number of bacteria quantified by serial dilution in phosphate buffered saline (PBS) buffer and expressed as CFU/mL and log_10_. The threshold value for an established skin infection by *S. aureus* was 10^5^ CFU/mL. All experiments were carried out 3–5 times. A reduction in the number of CFU/ml of 6 log_10_ was considered indicative of bactericidal activity (74).

### Therapy Protocols

Methylene Blue MB-aPDT treatment protocol on *S. aureus* infected skin had been compared with treatments based on Mupirocin, either alone (MU) or in combination (MB-aPDT + MU).

**Figure 2:**
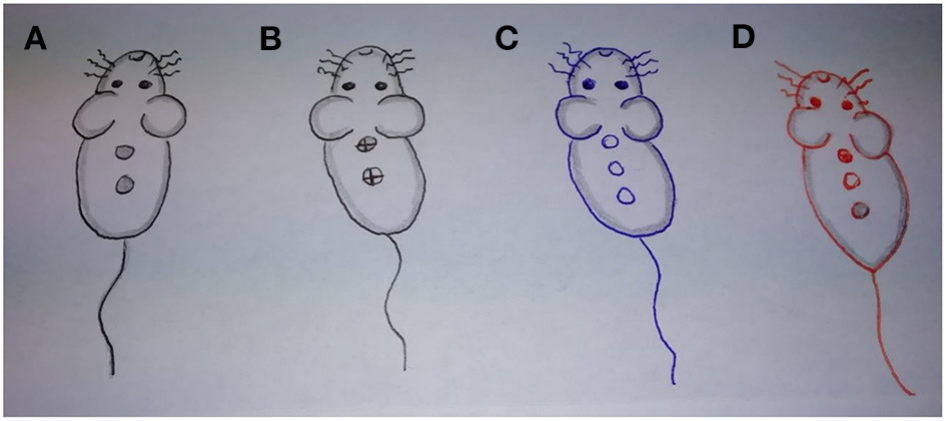
Sampling of murine wounds (handmade) for comparisons. **(A)**. Infected wounds treated with MB-aPDT. **(B)** Infected wounds treated with MU (dorsally combined with aPDT and ventrally in solitary). **(C)** Control (untreated) Infected wounds. **(D)** Healthy skin (dorsally ulcer by abrasion, in the middle intact skin, ventrally intact skin with MB).

In order to perform the tests, a total of 14 mice received wounds and were infected with *S. aureus*. Wounds were treated with the different protocols. Figure 2 shows the test distribution. As per control, some wounds infected were left untreated whilst some wounds were left uninfected.

Out of the 16 mice, 12 received 2 wounds whilst the remaining 4 mice received 3 wounds. By sampling in this way, we ensured that each mouse would act as control for its own treatments. Each mouse had two wounds infected excluding control (untreated) and healthy groups with three wounds for comparison purposes.

Since each of the 14 mice had two infected wounds, the testing was carried out comparing two treatment protocols until clinical resolution at each mouse. All possible combinations were tested: MB-aPDT and MU, MB-aPDT and MB-aPDT+MU, MB-aPDT and untreated, MB-aPDT+MU and MU, MB-aPDT and untreated, MU and untreated.

In total, 36 wounds were performed, out of which 12 received MB-aPDT treatment, 6 received MU treatment, 6 received a combination (MB-aPDT+MU), 6 were left untreated and 6 were not infected (Figure 2).

MB-aPDT treatment consisted of light irradiation of the infected wounds after sensitization with MB (Sigma-Aldrich® Corp., St. Louis, USA; Powder ≥ 82%; Absorption at 620–700 nm). MB was prepared in the dark just before use as previously reported (32, 74). Stock MB solution was prepared and diluted in bidistilled water to the desired concentration. Two drops of MB were deposited on the infected wounds and wounds were covered with a sticking plaster (Omnifilm®, Hartman) until complete reabsorption. Irradiation was started after 30 min of incubation in darkness (Figure 1C) (40). Under inhalated anesthesia, each wound was illumined with a light emitted diode (LED) lamp (Aktilite®, Photocure ASA, Oslo, Norway; fluence 74 J/cm^2^, 635 nm) for 16 min, at a distance of 6–8 cm (Figure 1D). A transient mild erythema was observed after irradiation.

MU treatment consisted on a daily application of ~0.1 mL of MU ointment (Mupirocin Isdin® 20 mg/gr, Barcelona, Spain) to the wound. An individual swap was used for each application and a gentle massage was applied until complete absorption of the ointment.

Combined treatment with MB-aPDT and MU consisted of a daily MU treatment applied, as previously described, started 24 h after the light irradiation of the MB-aPDT.

### Clinical Observations

Mice were monitored daily to assess health (mental and physical status) in response to the infection and the different therapies. Pathological characteristics of the wounds were assessed by the same investigator. The progression of the wound, estimating the days to get a reduction of 50% of size and the loss (detachment) of the crust (LC, time in days required to fall off) were evaluated. Additionally, gross lesions such as erythema and the presence of suppurative exudate, desquamation, contraction of the wounds and scars was also considered. Erythema and scaling were evaluated with visual skin-response scoring system as follows: 0 No observable effect; (1) Mild erythema; (2) Moderate erythema; (3) Strong erythema; (4) Dry desquamation; (5) Thin scab formation; (6) Thick scab formation (44). Digital images (Canon PowerShot A630©) and a caliper were utilized for quantitative assessment of wound lesions.

### Histopathological Studies

Biopsies were obtained by full-thickness excision of the skin at the time points of 24, 48, 76, and 96 h post-infection, and at the end of the experiment, when clinical healing was observed. Additionally, a sample of healthy skin was taken from each mouse to use as control. Samples were fixed in 10% phosphate buffered formalin solution and routinely processed. Briefly, tissues were embedded in paraffin, sectioned at 4 μm, stained with hematoxylin and eosin, and microscopically studied with a Nikon microscope (Axioskop 40). Photographs were taken with a camera (AxioCam MRa5) and morphometric analyses were performed with the AXIOVision Rel.5.6 software.

The assessment of the epidermal thickness is not very difficult to perform with routine stains because its border with dermis is sharp. On the other hand, dermis study was made by measuring up to the *panniculus carnosus* muscle. Although this includes the hypodermis that was severely affected by the inflammation induced by the experimental injury and differentiation between dermis and hypodermis was often not very clear. Immunohistochemistry staining that has allowed a much better assessment of the dermis, unfortunately, we were unable to perform.

Skin biopsies were studied in a blind fashion, without knowing the different treatments. Initially, the thickness of the epidermis and dermis were estimated. At the epidermis, the main lesions studied were the presence and thickness of hyperkeratosis (ortho-keratotic or parakeratotic), crusts and rete ridges. In the dermis, the severity of fibroplasia, with estimation of the density of fibrocytes and fibroblasts, follicular lesions (with special attention to granulomatous folliculitis due to its normal presence in this kind of mouse), dermatitis (superficial or deep) and panniculitis were studied. The increase in conjunctive tissue, the cellularity, the rete ridge and the follicular cyst size and number were evaluated in a semi-quantitative way establishing four categories: absent, mild, moderate, and severe.

### Statistical Analysis

All statistical analysis were performed with the SPSS software v.22 (IBM Corp., Armonk, NY, USA). Kolmogorov-Smirnov test was performed to assess of normality of data. Qualitative changes were evaluated by Chi Square and Cramer's V (significance V > 0.3). For non-parametric data, the Man-Withney or Kruskal-Wallys test was used to detect significant differences between groups (*p* < 0.001). Normal data were compared with no paired Student *t* or ANOVA test. Scheffe test was used for multiple comparison data *post-hoc*. *P* < 0.05 was considered as significant.

## Results

### Development of a Mouse Model of Skin Infection (Abrasive Wound Infection Model)

**Figure 3:**
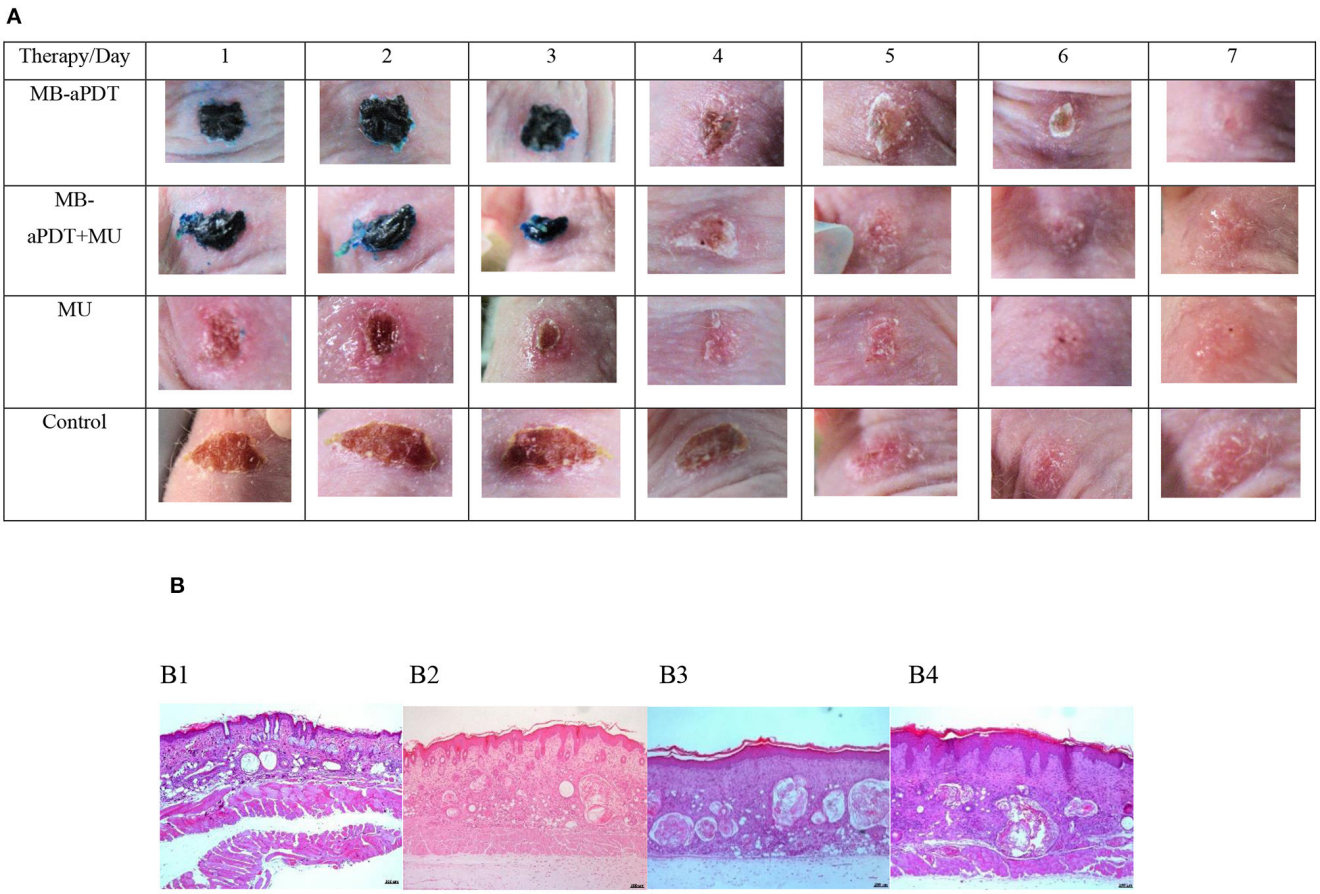
Comparison of photographs **(A)**/microphotographs **(B)**. **(A)** Clinical evolution of wounds infected by S. aureus during 7 days on therapeutical challenge and **(B)** healthy skin and infected wounds in function of therapy. **(A)** Photographs. **(B)** Microphotographs. From the left to the right of the image. (B1) Normal skin showing the mild undulation of the epidermis, thin dermis, dilated empty follicles typically observed in nude mice, and the muscle layer. H-E. x50. (B2) aPDT-treated wound. Note the mild acanthosis and mild undulation of the epidermis, a thicker dermis with moderate dermal fibrosis and more dilated follicles with abundant keratin and granulomatous inflammation. H-E. x50. (B3) aPDT+MU-treated wound. Epidermis shows slightly more acanthosis and orthokeratotic hyperkeratosis, slightly more cellularity in the dermis and more follicular reaction than aPDTtreated wounds. H-E. x50. (B4). MU-treated wound. Note intense acanthosis with abundant rete pegs and orthokeratotic hyperkeratosis, a slightly thinner dermis and a marked follicular reaction. H-E. x50.

Clinical evaluation, gross, and histopathological examinations, and determination of the bacterial burden in the wound demonstrated that *S. aureus* grew within the following 48 h post-inoculation reaching its maximum at this time point, when the bacterial count was ≥ 10^5^ CFU/mL and the natural healing occurred at 10–12 days. Clinical lesions resembled impetigo and were characterized by erythema, edema, and purulent exudate at 24 h post-infection (Figure 3). Large purulent crusts developed and corresponded with the peaking of bacterial burden at 48 h (Figure 1B). Wound contraction started by 72 h post-inoculation histologically corresponding with presence of epidermal micro-abscesses. At 96 h post-infection, macroscopic and microscopic hyperkeratosis were extensive. At 10–12 days post-infection natural clinical recovery was observed presenting equally skin hyperkeratosis or aberrant scars (data not shown). The histopathological study demonstrated the thickening of all cutaneous layers in comparison with healthy skin with a remarkable ortho-keratotic hyperkeratosis, acanthosis and dermal fibrosis (Figure 1E).

### Therapeutical Assay in *S. aureus* Infection Model

A summary of the clinical, microbiological and histological changes induced by the different treatments are listed in order of the magnitude of the effect in Table 1. Significant effects were demonstrated with all therapeutic approaches in our model of cutaneous *S. aureus* infection, in terms of clinical healing, cosmetic result (Table 2), histopathological events, and microbial burdens.

**Table 1:** Ranking of the different treatments for the statistically clinical, cosmetic, histological, and microbiological variables.

**Significant benefits of therapy**	
**Clinical healing**	
Size	
Size reduction	aPDT>MU>aPDT-MU>Control
50% Size reduction	aPDT>aPDT-MU>MU>Control
Crust Loss	aPDT<aPDT-MU<MU<Control
Purulence	aPDT<PDT-MU
Contraction	aPDT (24 h)
Erythema	aPDT<aPDT-MU
Clinical cure (days)	aPDT<aPDT-MU<MU<Control
**Cosmetic results**	
Scarless	aPDT<aPDT-MU=MU<Control
**Histopathology findings**	
Thickness hyperkeratosis	MU<aPDT<aPDT-MU
Dermis thickness	MU<aPDT<aPDT-MU
Size follicular cyst	MU<aPDT-MU<aPDT
**Microbial count**	
Log_10_	MU<aPDT-MU<aPDT<Control

*aPDT, antimicrobial photodynamic therapy; MU, mupirocin.*.

**Table 2:** Significant *p*-value and (V-Cramer) for qualitative aspects of wounds on therapy compared with untreated infected wounds.

**Parameter**	**Purulence**	**Erythema**	**Healed aspect**	**Scaling**	**Scarless**
**Treatment**					
aPDT	0.0002 (0.398)	<0.0001	0.0005 (0.402)	0.0329	<0.0001 (0.425)
aPDT + MU	0.0108	0.0429	0.0449	–	–
MU	–	–	–	<0.0088	–

*Chi Square/Fisher Exact p-value. (Table shows only significative V Cramer: v > 0.3)*.

*aPDT, antimicrobial photodynamic therapy; MU, mupirocin*.

#### Clinical Results

Wounds on therapy healed significantly before than wounds untreated: 7.18 (SD 1.00) days for MB-aPDT; 9.33 (SD 1.86) days for MU and also for the combination, and finally 10.33 (SD 1.0) days for controls (*p* < 0.001). When the different therapies were compared, MB-aPDT was statistically significant better than the combination (*p* = 0.001) or MU alone (*p* = 0.041).

The smallest size of the wound was achieved with MB-aPDT (0.21 ± 0.11 mm), followed by MU (0.33 ± 0.15 mm) and finally the combination (0.37 ± 0.17 mm) and all of them significantly smaller than without treatment. The size of the wound is one of most significant parameters correlated with others (Table 3) and also the microbial burden showing differences among therapies (Table 4). In the *post-hoc* comparisons, MB-aPDT was significantly better in terms of decreasing the size of the wound than MU and the combination (Table 4). Regarding the mean number of days for crust loss, this was significantly lower with MB-aPDT (4.69 ± 0.70) than with any other treatment (*p* < 0.001). It is surprising that the number of days for crust loss was significantly higher with MU (5.94 ± 1.24) than without any treatment (5.17 ± 1.07) (*p* = 0.44).

**Table 3:** Significant correlations (Pearson coefficient) and *p*-values and in numerical parameters for wounds in model assay (control) and wounds on therapy.

Correlations Pearson Coeficient^*^(*p*-value)	**Control**	**aPDT**	**aPDT + MU**	**MU**
Size vs. log_10_ bacteria	0.7^a^ (<0.0001)	0.48 (<0,0001)	0.58 (<0.0001)	0.6^a^ (<0.0001)
Size vs. Crust loss	0.44 (<0.0001)	0.46^b^ (<0.0001)	0.19 (0.0360)	0.3 (<0.0001)
Crust loss-SR50%	0.33 (0.0048)	0.39^b^ (<0.0001)	0.28 (0.0015)	–

*Measures: Size wounds (cm); Log_10_ (CFU/ml); Crust loss (CL. days): SR 50%: size reduction to 50% original (days)*.

* *Pearson coeficient: High: > 0.7; Moderate: 0.3–0.7; Mild < 0.3*.

a *Antimicrobial and size of wound*.

b *Wound healing*.

*aPDT, photodynamic therapy; MU, mupirocin*.

**Table 4:** Multiple comparison of quantitative effects of the different treatment vs. untreated wounds.

**Parameter (*p*-value)**	**Treatment**	***N***	**Mean ± SD**	**Significant Comparisons**	P*-value
Final microbiological count (Log_10_) (NS)	aPDT aPDT + MU MU Control	52 56 36 55	1.464 ± 1.740 0.950 ± 1.552 0.677 ± 1.346 1.711 ± 2.124	MU vs. aPDT aPDT+MU vs. control MU vs. control	0.0250 0.0332 0.0110
Size (cm) (< 0.0001)	aPDT aPDT + MU MU Control	52 56 36 55	0.208 ± 0.113 0.371 ± 0.169 0.328 ± 0.145 0.430 ± 0.105	aPDT vs. aPDT+MU aPDT vs. MU aPDT + MU vs. control aPDT vs. control MU vs. control	< 0.0001 < 0.0001 0.0287 < 0.0001 0.0002
Crust loss (days) (< 0.0001)	aPDT aPDT + MU MU Control	52 56 36 78	4.692 ± 0.701 5.643 ± 1.069 5.944 ± 1.241 5.167 ± 1.074	aPDT vs. aPDT + MU aPDT vs. MU aPDT+MU vs. control aPDT vs. control MU vs. control	< 0.0001 < 0.0001 0.0124 0.0058 0.0009
Size reduction 50% (days) (< 0.0001)	aPDT aPDT + MU MU Control	52 56 36 78	5.327 ± 0.706 7.125 ± 1.466 8.472 ± 2.569 10.667 ± 1.898	aPDT vs. aPDT+MU MU vs. aPDT + MU aPDT vs. MU aPDT + MU vs. control aPDT vs. control MU vs. control	< 0.0001 0.0019 < 0.0001 < 0.0001 < 0.0001 < 0.0001

* *Kruskal Wallis (p significative). aPDT, antimicrobial photodynamic therapy; MU, mupirocin*.

Regarding the qualitative aspects of the wounds infected with *S. aureus* on therapy (Table 2), MB-aPDT induces less purulent scabbing either in number of samples (24 vs. 76%) and in the size (0% exuberant vs. 20%) than the rest of the treatments (*p* = 0.44); additionally, the erythema was less frequent, being present in 13% of the lesions treated with MB-aPDT vs. in 87% of the wound receiving other treatments (*p* < 0.44). In fact, the most remarkable difference was in erythema, present until the third day in all wounds, but being more intense in those treated with MU alone than in the wounds with MB-aPDT or the combination (Figure 3). Regarding the presence of scaling, it was only seen in 36% of the lesions treated with MB-aPDT *vs*. in 60% of the aPDT-untreated (*p* = 0.44).

The most outstanding qualitative effect in the lesions treated exclusively with MU was the presence of scaling in 70% of the lesions, intense in 50% of them (*p* = 0.44) (Table 2). The combination of both treatments (MB-aPDT + MU) significantly reduced the erythema, present in 1 out of 4 lesions (25%) *vs*. in 75% of the lesions treated with MU and 45% of the untreated wounds (*p* = 0.44). The combination also reduced purulent scabs, absent in 77% of those treated with it and also from the qualitative point of view (0% of exuberant cases vs 15% in the untreated group) (*p* = 0.44). Finally, the combination also was superior to MU alone in terms of healing proportion (32% *vs*. 15%, *p* = 0.44). MU is the therapy that perpetuate crusts more even than untreated wounds in contrast to MB-aPDT (Table 4).

MB-aPDT is the treatment that cause scarfree benefits (Table 2) when scar was absent in 85% of the wounds treated with it comparing to presence of scar in 53% (43% hypertrophic) in those receiving MU or the combination (Figure 3).

#### Microbiological Results

MU (0.677-log_10_ ± 1.346) and the combination of MB-aPDT + MU (0.950-log_10_ ± 1.552) achieved the lowest microbiological count without statistically significant differences between them (Table 4). MU was superior than MB-aPDT (1.464-log_10_ ± 1.740; *p* = 0.025) in terms of microbiological reduction.

#### Histological Results

**Figure 4:**
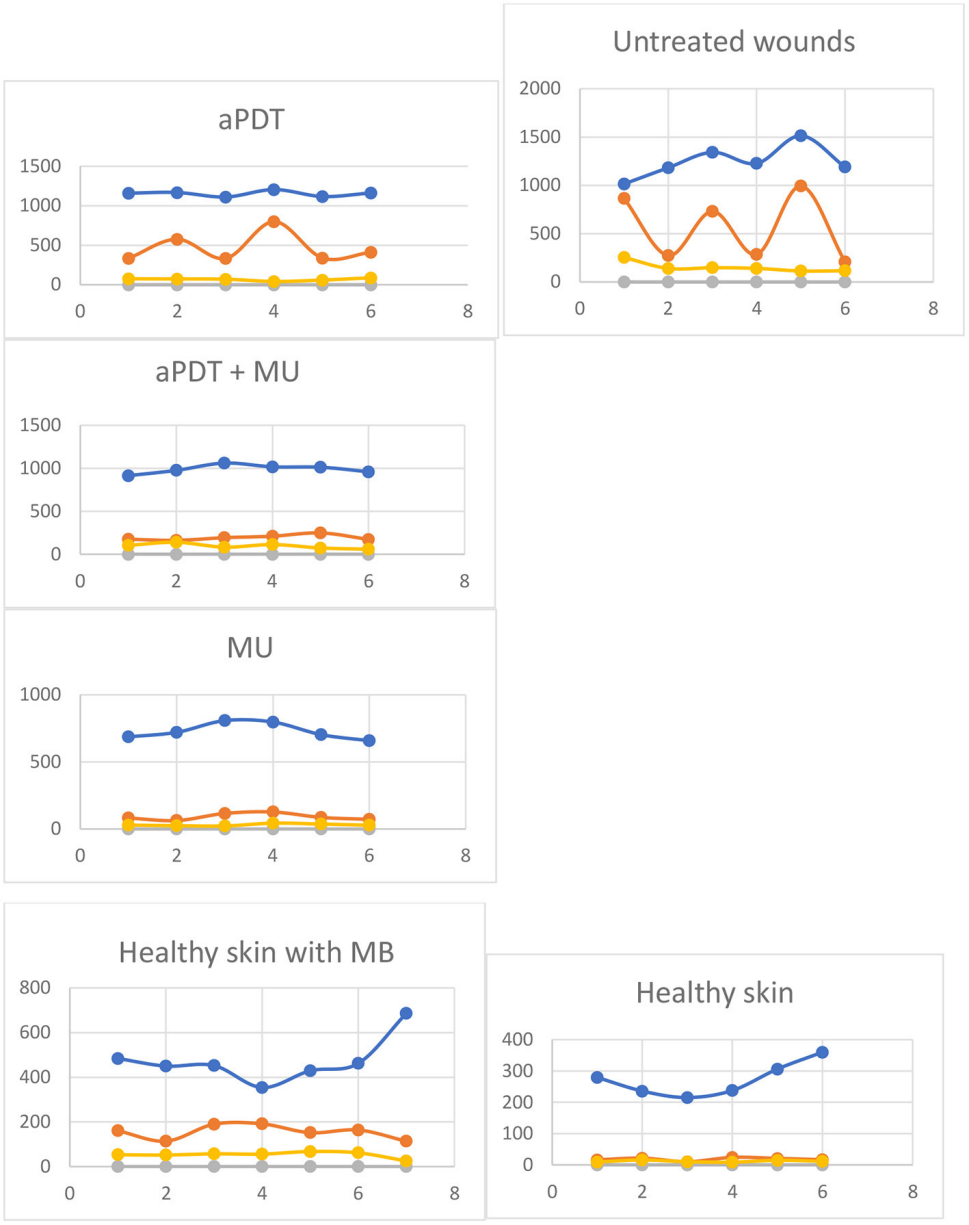
Dispersion of thickness of epidermis, dermis, and hyperkeratosis (Table 5) in wounds of SKH-1 mice on therapy or healthy skin. Blue: Dermis. Orange: Epidermis. Yellows: Hyperkeratosis. Vertical Axis: Thickness μm (mean ± SD). Horizontal axis: number of data.

Figure 1 shows the histological differences between the normal skin of the mice and the skin of the wound after spontaneous healing or the treatments. A remarkable increase in the thickness either of the epidermis or the dermis is observed (Figure 1E). Comparing the histological events induced by the different treatments, the thickness of the skin layers was lower with MU, followed by MB-aPDT and finally by the combination than of controls (*p* = 0.007 for dermal thickness) (Table 5). Dermal fibrosis and thickening were more present in wounds treated with MB-aPDT compared with a more intense epidermal reaction (acanthosis, rete ridges), hyperkeratosis and follicular cysts in those treated with MU alone (Figure 3B). In fact, the percentage of samples with intense increase in connective tissue and cellularity in the dermis was achieved with MB-aPDT (50%), even though the differences were not statistically different (Table 5). The increase in the size and also the number of follicular cyst size was more frequent in those samples treated with MU than with the other treatments, being the differences in the follicular cyst size statistically significant (*p* = 0.033). Variability of histological data on skin of SKH-1 mice are illustrated in Figure 4.

**Table 5:** Histologic findings of wounds on therapy against untreated and healthy skin.

**Level of Increase and wound examined**	**aPDT *N* (%)**	**aPDT+MU *N* (%)**	**MU *N* (%)**	**No treatment**	**Healthy skin**	***P*-value**
Conjunctive tissue						0.951
-Mild -Moderate -Intense	2 (33.3) 1 (16.7) 3 (50.0)	2 (40) 2 (40) 1 (20)	0 (0) 5 (83.3) 1 (16.7)	1 (50) 0 (0) 1 (50)		
Cellularity						0.695
-Mild -Moderate -Intense	1 (16.7) 2 (33.3) 3 (50)	2 (40) 2 (40) 1 (20)	0 (0) 5 (83.3) 1 (16.7)	1 (50) 1 (50) 0 (0)		
Rete ridge						0.430
-Mild -Moderate -Intense	2 (40) 2 (40) 1 (20)	3 (75) 1 (25) 0 (0)	0 (0) 4 (66.7) 2 (33.3)	1 (50) 0 (0) 1 (50)		
Follicular Cyst size						0.033^*^
-Mild -Moderate -Intense	2 (33.3) 3 (50) 1 (16.7)	2 (33.3) 1 (16.7) 3 (50)	0 (0) 2 (33.3) 4 (66.7)	1 (50) 1 (50) 0 (0)		
Follicular Cyst number						0.111
-Mild -Moderate -Intense	3 (50) 2 (33.3) 1 (16.7)	2 (33.3) 1 (16.7) 3 (50)	1 (16.7) 2 (33.3) 3 (50.0)	1 (50) 1 (50) 0 (0)		
Epidermis Thickness (μm) (mean, SD)	230.51 (166.38)	209.13 (120.20)	122.99 (78.89)	370.82 (266.92)	18.93 (1.04)	0.118
Dermis thickness(μm) (mean, SD)	866.71 (217.90)	918.09 (217.88)	706.09 (69.01)	1092.14 (216.53)	346.39 (104.73)	0.007^*^
Hyperkeratosis thickness (μm) (mean, SD)	44.34 (36.75)	63.71 (39.98)	33.37 (8.52)	152.16	8.76 (3.98)	0.013^*^

* *p < 0.05 significative; aPDT, antimicrobial photodynamic therapy; MU, mupirocin*.

## Discussion

The current study shows that the three treatment protocols tested, MB-aPDT, MU, and MB-aPDT +MU, were beneficial compared to self-healing; whereas mupirocin showed the higher logaritmic reduction of *S. aureus*, MB-aPDT was better in the speed wound healing and cosmetic result. The experimental model of abrasive superficial *S. aureus-*infection wound in SKH-1 hairless mice is not useful enough for pre-clinical studies to establish the efficacy of antimicrobial treatments, although its self-healing condition could have limited the evaluation of synergistic effects in our study.

Our abrasive skin wound is more similar to real infection than *tape stripping* (45) and needs a higher bacterial inoculum (21, 34). Clinically, is very similar to human impetigo, with a purulent infection established in 24 h, dermis affection and pyo-granuloma during maturation of *S. aureus* at 48 h, epidermal purulent micro-abscess (72 h) and contraction and large crust of purulent material with histopathological hyperkeratosis (96 h) (18, 46, 47). Wounds healed *per se* appear with significative thickening of skin layers and dermal epithelial cysts, typical of this hairless mouse model, and clinically with desquamation and hypertrophic scar (16, 23, 48).

MB-aPDT was better than the treatment with MU in terms of healing speed of the wound and cosmetic result. This reduction of size of wound cited by Topaloglu et al. (49) and crust loss in days, shows evident differences in favor of aPDT as described by Dai et al. (34). Not only changes in size wound but also significative contraction in the first day after MB-aPDT in comparison with 72 h of untreated wounds. By contrast, MU treatment achieves the highest microbiological reduction. This apparent discordance between microbiological and clinical healing has been previously reported in Wistar rats, where topical MU shows efficacy against wound infection inoculated with *S. aureus* (50). Furthermore, the count of bacteria from infected mice may be inconsistent (51) and for that reason, other methods can be recommended in addition as bioluminescent monitoring (52) or the culture of supernatant of skin (47).

Regarding the combination of both treatments, MB-aPDT + MU was neither superior than MB-aPDT, in terms of clinical healing of the infected wound, nor than MU, in terms of microbiological cure, and its results seem to be nearest from MU than from MB-aPDT. Therefore, the combination did not seem to have a synergistic effect in contrast to what is shown in the *in vitro* study performed by our group (53). One explanation could be that in the *in vitro* and *in vivo* concentration of MU used were not the same and neither the bactericidal effect. *In vivo* experiment was carried out using the concentration of MU used to treat cutaneous infections (54), while in the *in vitro* study, the concentration of MU used did not significantly reduce the bacterial load by itself (53). Other possible explanation is the disadvantage of our animal model in which the infected wounds spontaneously healed in 10–12 days. To our knowledge, there are not murine studies using aPDT combined with MU in infected abrasive wounds. However, there are accelerated healing infected assays recently described (55, 56). Besides, most of the studied combinations using aPDT + antimicrobial tried so far were not designed about approved standards, making difficult to compare the results (38, 40, 57).

The good results in the scarring process shown by MB-aPDT (alone or combined vs. MU) correlates with the presence of more connective tissue in histology and also cellularity in the dermis (fibroblasts). It has been shown that aPDT induces fibroblasts proliferation and, consequently, an increase of collagen and elastin with better healing activity (58). Contraction in the chronology of the wound healing is an early event with MB-aPDT (24 h post-aPDT) that reduces the size because of the centripetal movement of the wound margins (50) even combined with MU. Reports are inconclusive, with Bairy et al. (59) who detected contraction in burns treated with MU in rats, whereas Erdur et al., did not observe any contraction (50). Experimental factors such as type of animal or cutaneous response could explain these contradictions.

Histopathology is the gold standard method to measure wound healing and to determine re-epithelialization of epidermis (43, 60–62). Skin of hairless mice, free of rete pegs, develops a pseudo-epitheliomatous hyperplasia during healing, similar to the human rete pegs, source of keratinocytes during skin healing (63). Rete pegs, in our study, were only present in MB-aPDT wounds besides a more conjunctive tissue response (Table 5) by hyperplasticity of epidermis in nude mice described in 1952 (48). Treatment reduces dermal response and histopathological hyperkeratosis, asynchrony dermis/epidermis (16) and increase of size of cysts, not referred before (Table 5). Infected abrasions treated showed lesser inflammatory findings during the MU treatment and more conjunctive response of aPDT as reported Jorge et al. (64) in nude mice without cyst changes, in contrast with our findings (Figure 3B4). Epidermis on therapy showed more hyperkeratosis than acanthosis (65) representing a recovery way of healing of this superficial model of *S. aureus* in nude mice (66).

Erythema, non-detachment of crust and bigger cyts are irritative effects during MU therapy, to our knowledge first described. Erythema, is the main component of purulent erythematous dermatitis in humans (28) and was present with all therapies, being more remarkable in MU treated wounds, although always milder than in untreated wounds. A previous report in a mouse model of wound infected with methicillin-resistant *S. aureus* shows the same degree of inflammatory infiltrate at 24 h of MB-aPDT as without treatment (67); we do not know how to explain this difference considering that 2% MU (Bactroban) has little or no potential for irritation in previous studies (54). In our opinion, erythema during treatment with MU is a clinical sign related with an irritative response more than a weakened inflammatory control as the histological study demonstrates (Table 5).

In the present study MU shows the worse clinical parameters of the three treatments in terms of the clinical evolution of the infected wound. In fact, delayed detachment of the crust was significantly abnormal for MU wounds, even more retarded than untreated wounds (Table 4). Speculations about unknown role of crusts in the microbial clearance of *S. aureus* (67) does not explain this delay in MU wounds because the microbial reduction was the highest. An increase of tissue force with higher resistance of the scar due to organization of collagen may justify this detected delay (68). Without evident detrimental effects on healing (69), MU could injure keratinocytes and fibroblasts of healthy skin (68) what stimulates new formulations (70) or synergistic antimicrobial combinations with best healing properties (71). Hyperkeratosis/clinical desquamation in MU-wounds shows clinical-histological correspondence with an excess of keratin production and superficial cutaneous desquamation in nude mice (23); this could have a double explanation: uncontrolled wound healing in untreated wounds or changes of lipid layer of skin as secondary effect of repetitive dose of topical MU in humans (drug commercial package) and possibly in SKH-1 mice. The last significative finding due to repetitive administration of MU ointment on SKH-1 mice is larger follicular cyst, to our knowledge, described for the first time (Table 5).

Our clinical results agree with the already reported beneficial effect of aPDT on healing of *S. aureus* infected wounds in other models (34, 49, 72, 73, 77) and also in human patients (75). Furthermore, our clinical results add evidence about that MB-aPDT in particular improves the healing of different causal agent-infected skin wounds as previously was demonstrated by our group in sheep (74), recently in cattle (76) and it has been equally reported in humans (27).

Here, we present a complete correspondence of biological, clinical, and histopathological findings in a superficial abrasive model of *S. aureus* infection in SKH-1 mice. Chronology in clinical signs (78) of cutaneous events during therapy promotes contraction and detachment of crust as highlighted indexes of wound healing. Correlations as size lesion and bacterial count in this SKH-1 model (78), size lesion changes and healing during aPDT treatment (79) were corroborated. Self-limiting of this model (23), related with experimental factors (skin reparation), promotes the clearing of bacteria (10, 40); being a disadvantage to achieve a most robust model of skin *S. aureus* infection, on the other hand the lack of epidermis is an excellent model of repeated abrasion caused by human scratching (47). A more robust model of infection may find more synergistic results that those presented here. Antimicrobial failure and synergy response were cited (80, 81). Nevertheless, this model has two main limitations: first, the fact that even without treatment the infection and the wound cured in an immunocompetent model (47) although correlation of clinical events and histopathological findings are strong in staphylococcal infections (78), second, the lack of males, because both skin layer differences by gender have been described (18, 82). Additionally, biological variability of SKH-1 hairless mice (23) during wound healing (66) should be considered. These results in animals support to carry out clinical trials to use MB-PDT in infected wounds evaluating not only the microbiological clinical effect against *S. aureus* infections but also to stimulate wound healing (75).

## Conclusions

Clinical signs, gross observations, and histopathological findings are concurrent in this abrasive infection model. Superficial wounds on therapy with one session of MB-aPDT have shorter clinical duration, contraction with best healing and good recuperation, whereas MU-wounds achieve the best antimicrobial/anti-inflammation control. The increase of size of follicular cyst on the wounded skin of SKH-1 mice and skin asynchrony that produces therapies are significative, considering variability of data in nude mice. We did not found benefits in combining MB-aPDT + MU in our experimental model of wound superficial infection with *S. aureus* in SKH-1 hairless mice; further studies, in a model without the capacity of self-resolution of the infected wound and also using MU resistant *S. aureus* strains, are needed in order to confirm these results and discard the possible usefulness of this combination. However, this study provides clear evidence on the usefulness of the MB-aPDT, so it can help to support its use in the clinic and we hope that it will help to extend its antimicrobial and healing application. Its main advantage arises from being an alternative treatment without using antibiotics, therefore it will not contribute to the selection of resistant strains, and the efficacy is independent of the pattern of antimicrobial resistance of the strains implicated in the infection. Skin benefits has been demonstrated for healing use of MB-aPDT in clinical practice.

## Data Availability Statement

The raw data supporting the conclusions of this article will be made available by the authors, without undue reservation.

## Ethics Statement

The animal study was reviewed and approved by Ethic Committee for Animal Experiments from the University of Zaragoza (PI40/13).

## Author Contributions

MP and MV participated equally in the design, conducted all animal experiments, and performed and draft this manuscript. PR develop microbiological procedures and participated in the design. YG, AR, and CA participated equally at the design of experiment, statistically results, and design of these experiments. RB and BM elaborated histopathological procedures and its interpretation. VP-L contributes with his knowledge on *S. aureus* and MB-aPDT. All authors read and approved this manuscript.

## Conflict of Interest

The authors declare that the research was conducted in the absence of any commercial or financial relationships that could be construed as a potential conflict of interest.
